# Supplementary material for: Optimal Localist and Distributed Coding of Spatiotemporal Spike Patterns Through STDP and Coincidence Detection
Source: Front Comput Neurosci. 2018 Sep 18;12:74. doi: 10.3389/fncom.2018.00074 (PMC6153331; doi:10.3389/fncom.2018.00074)
Supplement: Supplementary file 1 [file Data_Sheet_1.pdf]

Optimal localist and distributed coding of spatiotemporal spike patterns through STDP and coincidence detection

Timothée Masquelier & Saeed Reza Kheradpisheh

Appendix

Graded weights

In this paper, we assumed unitary (or binary) synaptic weights: all connected afferents had the same synaptic weight[1]. This constraint strongly simplified the analytical calculations. But could the SNR be even higher if we removed this constraint, and by how much? Intuitively, when one wants to detect a spike pattern that has just occurred, one should put strong weights on the synapses corresponding to the most recent pattern spikes, since these weights will increase $V_{\max}$ more than V_{noise}. Conversely, very old pattern spikes that fall outside the integration window (if any) should be associated to nil weights: any positive value would only increase V_{noise}, not $V_{\max}$. But between those two extremes, it might be a good idea to use intermediate weight values.

To check this intuition, we used numerical optimizations using a simplified setup. We used a single pattern ($P = 1$), that was repeated in the absence of jitter ($T = 0$). We divided the pattern into n different periods $\Delta t_1, ...\Delta t_n$ (in reverse chronological order), each one corresponding to a different synaptic weight $w_1, ...w_n$ (see Figure 1 left for an example with $n = 2$). More specifically: the M_1 afferents that fire in the Δt_1 window are connected with weight w_1. The M_2 afferents that fire in the Δt_2 window, but not in the Δt_1 one, are connected with weight w_2. More generally, the M_i afferents that fire in the Δt_i window, but not in the $\Delta t_1 ...\Delta t_{i-1}$ ones, are connected with weight w_i.

With this simple set up, the SNR can be computed analytically. For example, if $n = 2$ (Fig. 1 left), we have:

$$\langle M_1 \rangle = N(1 - e^{-f\Delta t_1}), \tag{1}$$

$$\langle M_2 \rangle = N(1 - e^{-f\Delta t_2})e^{-f\Delta t_1}. \tag{2}$$

The asymptotic steady regimes for the two time windows are:

$$\langle V_1^\infty \rangle = \tau f w_1 N, \tag{3}$$

$$\langle V_2^\infty \rangle = \tau f \left(w_2 N + (w_1 - w_2) \langle M_1 \rangle \right). \tag{4}$$

Let's call V_i the potential at the end of window Δt_i, and $V_{n+1} = V_{\text{noise}}$. Then $V_{\max} = V_1$ can be computed iteratively:

$$V_2 = (1 - e^{-\Delta t_2/\tau})(V_2^\infty - V_3), \tag{5}$$

$$V_1 = (1 - e^{-\Delta t_1/\tau})(V_1^\infty - V_2). \tag{6}$$

Furthermore [Burkitt, 2006],

$$V_{\text{noise}} = \tau f (w_1 M_1 + w_2 M_2), \tag{7}$$

and:

$$\sigma_{\text{noise}} = \sqrt{\tau f (w_1^2 M_1 + w_2^2 M_2)/2}. \tag{8}$$

So we have everything we need to compute the SNR.

Equations 1 – 8 can be generalized to $n > 2$:

$$\langle M_i \rangle = N(1 - e^{-f\Delta t_i})e^{-f\sum_{j=1}^{i-1}\Delta t_j}, \tag{9}$$

$$\langle V_i^\infty \rangle = \tau f \left(w_i N + \sum_{j=1}^{i-1} (w_j - w_i) \langle M_j \rangle \right) \tag{10}$$

and $V_{\max} = V_1$ can be computed iteratively from $V_{n+1} = V_{\text{noise}}$ using:

$$V_{i-1} = (1 - e^{-\Delta t_{i-1}/\tau})(V_{i-1}^\infty - V_i). \tag{11}$$

Furthermore [Burkitt, 2006],

$$V_{\text{noise}} = \tau f \sum w_i M_i, \tag{12}$$

[1] Numerical simulations with STDP used graded weights during learning, but not after convergence.

and:

$$\sigma_{\text{noise}} = \sqrt{\tau f \sum w_i^2 M_i / 2}. \tag{13}$$

So the SNR can be computed for any n, and, importantly, it is differentiable with respect to the w_i. We can thus use efficient numerical methods to optimize these weights. Since scaling the weights does not change the SNR, we imposed $w_1 = 1$. Figure 1 right gives an example with $n = 70$. Here the Δt_i were all equal to $5\tau/n$, and we optimized the corresponding w_i. We chose $\tau = 10$ms, and $f = 1$, 5, and 10Hz. The gain w.r.t. binary weights for the SNR were modest: 10.5%, 9.6% and 8.9% respectively. As f tends towards 0, the optimal weights appears to converge towards $e^{t/\tau}$ (even if we could not prove it): the $f = 1$Hz curve (solid blue) is almost identical to $e^{t/\tau}$ (dashed red).

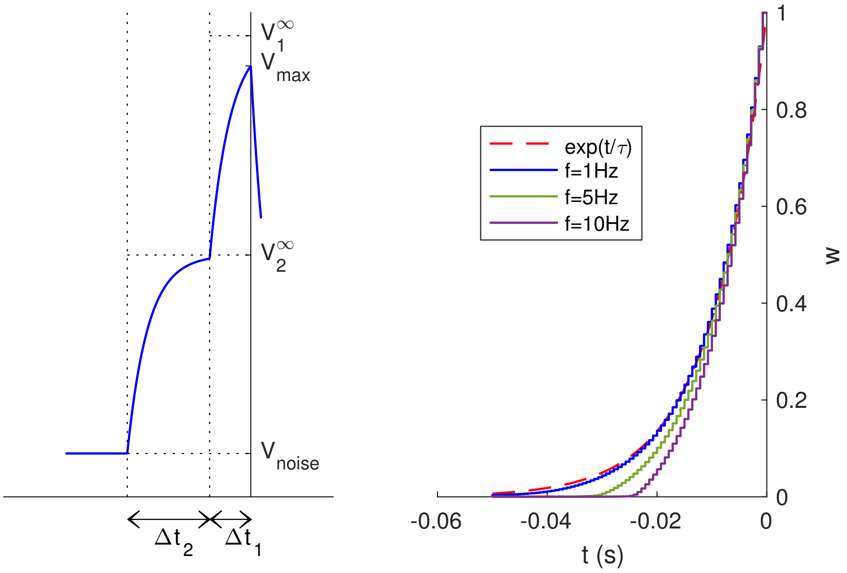

Fig. 1: Optimization with graded weights. (Left) Didactic example with $n = 2$ weight values: w_1 for all the afferents that fire in the Δt_1 window, and $w_2 < w_1$ for all the afferents that fire in the Δt_2 window but not in the Δt_1 one. V_1^∞ and V_2^∞ are the asymptotic potentials for the two periods. $V_{\max}$ can be computed from those two values (see text). (Right) Numerical optimization of the weights with $n = 70$. With small f, the optimal solution appears to be close to $e^{t/\tau}$.

References

Burkitt, A. N. (2006). A review of the integrate-and-fire neuron model: I. Homogeneous synaptic input. *Biological Cybernetics*, 95(1):1–19.
